# Carotenoid metabolism: New insights and synthetic approaches

**DOI:** 10.3389/fpls.2022.1072061

**Published:** 2023-01-18

**Authors:** Alice Stra, Lamyaa O. Almarwaey, Yagiz Alagoz, Juan C. Moreno, Salim Al-Babili

**Affiliations:** ^1^ The Bioactives Laboratory, Center for Desert Agriculture, King Abdullah University of Science and Technology (KAUST), Thuwal, Saudi Arabia; ^2^ Biological and Environmental Science and Engineering Division, King Abdullah University of Science and Technology (KAUST), Thuwal, Saudi Arabia

**Keywords:** apocarotenoids, biofortification, carotenoids, crop improvement, microorganisms, metabolic engineering, synthetic biology

## Abstract

Carotenoids are well-known isoprenoid pigments naturally produced by plants, algae, photosynthetic bacteria as well as by several heterotrophic microorganisms. In plants, they are synthesized in plastids where they play essential roles in light-harvesting and in protecting the photosynthetic apparatus from reactive oxygen species (ROS). Carotenoids are also precursors of bioactive metabolites called apocarotenoids, including vitamin A and the phytohormones abscisic acid (ABA) and strigolactones (SLs). Genetic engineering of carotenogenesis made possible the enhancement of the nutritional value of many crops. New metabolic engineering approaches have recently been developed to modulate carotenoid content, including the employment of CRISPR technologies for single-base editing and the integration of exogenous genes into specific “safe harbors” in the genome. In addition, recent studies revealed the option of synthetic conversion of leaf chloroplasts into chromoplasts, thus increasing carotenoid storage capacity and boosting the nutritional value of green plant tissues. Moreover, transient gene expression through viral vectors allowed the accumulation of carotenoids outside the plastid. Furthermore, the utilization of engineered microorganisms allowed efficient mass production of carotenoids, making it convenient for industrial practices. Interestingly, manipulation of carotenoid biosynthesis can also influence plant architecture, and positively impact growth and yield, making it an important target for crop improvements beyond biofortification. Here, we briefly describe carotenoid biosynthesis and highlight the latest advances and discoveries related to synthetic carotenoid metabolism in plants and microorganisms.

## Carotenoid biosynthetic pathway in plants and microorganisms

Carotenoids are essential hydrophobic compounds that participate in many aspects of plant’s life (Krinsky, 1989; Ruiz-Sola and Rodríguez-Concepción, 2012; Zulfiqar et al., 2021). They are essential constituents of the light-harvesting complexes, due to their involvement in harnessing light energy and capacity to quench chlorophyll triplets and to scavenge singlet oxygen (Mascoli et al., 2021). Carotenoids are also responsible for the yellow, orange and red colorations of several fruits and flowers, attracting insects and animals for pollination and seed dispersal (Tanaka et al., 2008; Yuan et al., 2015). Moreover, these pigments are the source of an important family of metabolites, called apocarotenoids, which includes retinal, abscisic acid (ABA) and strigolactones (SL) (Al-Babili and Bouwmeester, 2015; Felemban et al., 2019; Wang et al., 2019; Moreno et al., 2021a; Wang et al., 2021c; Jia et al., 2022). In plants, some apocarotenoids function as retrograde signals, triggering responses to adapt to several stress conditions, including high-light, high-salt and drought (Hou et al., 2016; Felemban et al., 2019; Wang et al., 2019; Moreno et al., 2020).

**Figure 1:**
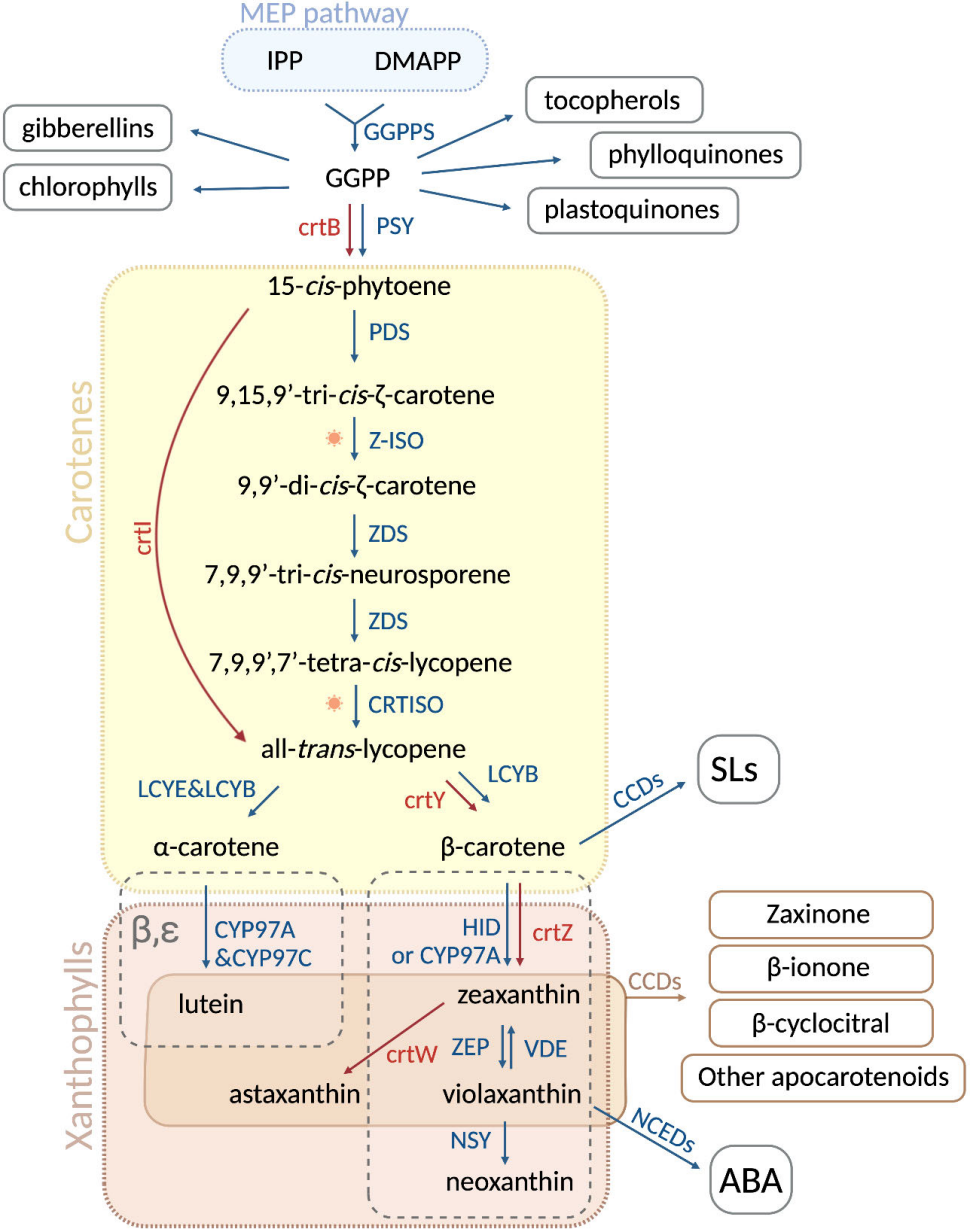
Carotenoid biosynthetic pathway in plants and microorganisms. In plastids, the condensation of isopentenyl diphosphate (IPP) and dimethylallyl diphosphate (DMAPP), derived from the MEP pathway, is catalyzed by GGPPS and gives rise to geranylgeranyl pyrophosphate (GGPP). The latter is a precursor of several important plastid isoprenoids, such as tocopherols, phylloquinones, plastoquinones, chlorophylls and gibberellins. Two GGPP molecules are then condensed into 15-*cis*-phytoene by PHYTOENE SYNTASE (PSY) in plants and by crtB in bacteria and fungi. Sequentially, the enzymes phytoene desaturase (PDS), ζ-CAROTENE ISOMERASE (Z-ISO), ζ-CAROTENE DESATURASE (ZDS) and CAROTENOID ISOMERASE (CRTISO) catalyze a series of desaturations and isomerizations, producing all-*trans*-lycopene from 15-*cis*-phytoene. In most fungi and bacteria, this conversion is carried out by a single enzyme, PHYTOENE DESATURASE (crtI). At this point, all-*trans*-lycopene undergoes a cyclization step, which is performed by LYCOPENE ϵ -CYCLASE (LCYE) and LYCOPENE β-CYCLASE (LCYB), leading to α-carotene and β-carotene, respectively. In fungi and non-photosynthetic bacteria, β-carotene is formed by crtY. In the α branch, the cytochrome P450 enzymes CYP97A and CYP97C convert α-carotene into lutein, while the non-heme diiron oxidase (HYD)/CYP97A in the β-branch transforms β-carotene into zeaxanthin. In microorganisms, the enzyme crtZ produces zeaxanthin, which can be further converted by crtW into astaxanthin. In plants, ZEAXANTHIN EPOXIDASE (ZEP) is responsible of the conversion of zeaxanthin into violaxanthin, which can be reversed into zeaxanthin through the action of VIOLAXANTHIN DE-EPOXIDASE (VDE). Violaxanthin is then transformed into neoxanthin by NEOXANTHIN SYNTHASE (NSY) as a last step of the pathway. Oxidative cleavage of carotenoids gives rise to apocarotenoids. The cleavage of β-carotene performed by carotenoid cleavage dioxygenases (CCDs) generates carlactone (not shown) the precursor of strigolactones (SLs), whereas 9-*cis*-violaxanthin is cleaved into the abscisic acid (ABA) precursor, xanthoxin (not shown), by nine-*cis*-epoxy-carotenoid dioxygenases (NCEDs). Plant and microbial enzymes are colored in blue and red, resperctively. Sun image represents photoisomerization. Figure was designed in Biorender.

Carotenoids are synthesized by all photosynthetic organisms and some heterotrophic fungi and bacteria (Fraser and Bramley, 2004; Cazzonelli and Pogson, 2010; Ruiz-Sola and Rodríguez-Concepción, 2012; Sun et al., 2022). In green organisms, carotenoid biosynthesis takes place in plastids, mediated by nuclear-encoded enzymes (Shumskaya and Wurtzel, 2013; Moise et al., 2014; Alagoz et al., 2018; Moreno et al., 2021a). Carotenoids are formed from isopentenyl diphosphate (IPP, C_5_) and its isomer dimethylallyl diphosphate (DMAPP, C_5_), both derived from the plastid methylerythritol (MEP) pathway (Ruiz-Sola and Rodríguez-Concepción, 2012). The condensation of one IPP with one DMAPP molecule, followed by sequential condensation with two IPP molecules, gives rise to geranylgeranyl diphosphate (GGPP, C_20_), a precursor of many plastid isoprenoids (Bouvier et al., 2005; Ruiz-Sola and Rodríguez-Concepción, 2012; Moise et al., 2014). Consequently, the condensation of two GGPP molecules produces the first carotenoid, the colorless 15-*cis*-phytoene (**Figure 1**). This reaction is catalyzed by the rate-limiting enzyme PHYTOENE SYNTHASE (PSY), called crtB in bacteria (Cunningham and Gantt, 1998). 15-*cis*-phytoene undergoes an array of sequential desaturations and isomerizations that increase the number of conjugated double-bonds from three to eleven. The first two-step desaturation reaction is catalyzed by PHYTOENE DESATURASE (PDS), producing 9,15,9’-tri-*cis*-ζ-carotene, which is then transformed into 9,9’-di-*cis*-ζ-carotene by ζ-CAROTENE ISOMERASE (Z-ISO) (Beltrán et al., 2015; Zhang et al., 2022). The activity of Z-ISO can be partially compensated by photoisomerization (Beltrán et al., 2015). Then, ζ-CAROTENE DESATURASE (ZDS) catalyzes another two steps of desaturation, giving rise to 7,9,9’-tri-*cis*-neurosporene and eventually to 7,9,9’,7’-tetra-*cis*-lycopene. Finally, CAROTENOID ISOMERASE (CRTISO) converts 7,9,9’,7’-tetra-*cis*-lycopene into all-*trans*-lycopene (**Figure 1**). Most bacteria and fungi can convert 15-*cis*-phytoene to all-*trans*-lycopene with a single enzyme, e.g. PHYTOENE DESATURASE (crtI) from *Pantoea ananatis* (Bartley and Scolnik, 1989; Armstrong et al., 1989; Schaub et al., 2012), or AL-1 from the ascomycete *Neurospora crassa* (Díaz-Sánchez et al., 2011). Lycopene is the precursor of cyclic carotenoids that carry different types of unmodified or modified ionone rings, such as β- or ε-ionone ring, at the ends of the linear chain (Cunningham and Gantt, 1998; Moise et al., 2014; Fernandes et al., 2018). These cyclization reactions mark the branching point of the pathway, thus generating carotenoid diversity based on the end groups and their modifications. In the α-carotenoid branch, LYCOPENE Ɛ-CYCLASE (LCYE) together with LYCOPENE β-CYCLASE (LCYB) produce α-carotene by introducing an ϵ- and a β-ionone ring, respectively. In the β-branch, the plant LCYB and the bacterial crtY form two β-ionone rings to yield β-carotene from lycopene (**Figure 1**) (Visser et al., 2003; Wang et al., 2021a). The hydroxylation of the β- and ϵ-ionone rings leads to xanthophylls. Cytochrome P450 enzymes CYP97A and CYP97C catalyze the formation of lutein from α-carotene, whereas non-heme diiron hydroxylase (HYD) and CYP97A are responsible for the conversion of β-carotene into zeaxanthin (Tian et al., 2004; Kim and DellaPenna, 2006; Quinlan et al., 2012). Lutein represents the end product of the α-carotenoid branch in green tissues, while zeaxanthin can be reversibly epoxidated by the ZEAXANTHIN EPOXIDASE (ZEP), yielding violaxanthin (Tian et al., 2004; Kim and DellaPenna, 2006; Quinlan et al., 2012). Violaxantin can then be converted back by the VIOLAXANTHIN DE-EPOXIDASE (VDE) (Rockholm and Yamamoto, 1996). The final step of the carotenoid pathway is the formation of neoxanthin from violaxanthin, catalyzed by the NEOXANTHIN SYNTHASE (NSY) (**Figure 1**). In microorganisms, crtZ converts β-carotene into zeaxanthin that can be transformed into astaxanthin by the ketolase crtW (Zhang et al., 2022).

Having a highly unsaturated hydrocarbon backbone makes carotenoids prone to oxidation (Ahrazem et al., 2016; Hou et al., 2016; Schaub et al., 2018; Wang et al., 2019; Koschmieder et al., 2021). This process can occur non-enzymatically when the conjugated double bonds are attacked by ROS (Moreno et al., 2021a), or catalyzed by highly specific CAROTENOID CLEAVAGE DIOXYGENASES (CCDs) and 9-*CIS*-EPOXY-CAROTENOID DIOXYGENASES (NCEDs) (Giuliano et al., 2003; Ahrazem et al., 2016; Hou et al., 2016). Oxidative cleavage of carotenoids yields bioactive apocarotenoids, including the precursors of the plant hormones ABA and SL, which are produced by NCEDs, CCD7 and CCD8, respectively (Sorefan et al., 2003; Booker et al., 2004; Johnson et al., 2006; Cutler et al., 2010; Felemban et al., 2019). Apocarotenoids exert a series of further biological activities, such as regulating of growth, biotic and abiotic stress response, retrograde signaling, photo acclimation, and include pigments and volatiles that play a role in plant-animal communication (Wang et al., 2019; Jia et al., 2019; Moreno et al., 2020; Moreno et al., 2021a). For instance, it has been recently shown that the apocarotenoid zaxinone is involved in the modulation of plant growth and in regulating SLs level and arbuscular-mycorrhizal (AM) colonization in rice (**Figure 1**), and SL and ABA levels in Arabidopsis (Wang et al., 2019; Ablazov et al., 2020; Votta et al., 2022). Moreover, the volatile apocarotenoid β-ionone, usually released by leaves, contributes to the scent of flowers in many plants and plays an interesting role as a herbivore repellent in plant-insect interaction (**Figure 1**) (Ômura et al., 2000; Gruber et al., 2009; Moreno et al., 2021a). Furthermore, β-cyclocitral (β-cc) is another volatile and bioactive apocarotenoid. β-cc mediates ^1^O_2_ signaling and tolerance against abiotic stresses, and its oxidation give rise to β-cyclocitric acid that increases salt and drought tolerance (**Figure 1**) (Ramel et al., 2012; D'Alessandro et al., 2019; Moreno et al., 2021a). In addition, it has been recently shown that β-cc is also a conserved root regulator (Dickinson et al., 2019).

A deep understanding of the carotenoid pathway in plants and microorganisms can provide new tools and open up new options for establishing synthetic metabolism of carotenoids and enriching them and their derivatives in different organisms. In this mini-review, we summarize recent findings and the latest approaches to engineer carotenoid synthesis in plants and microorganisms for biofortification and beyond.

## Carotenoid biofortification in plants

Generating biofortified crops is a long-term and worthwhile biotechnological goal to enhance the nutritional value of crops. Indeed, micronutrient malnutrition is still a significant public health problem that affects about one-third of the world’s population (Thompson and Amoroso, 2014). Therefore, several staple crops have been biofortified to accumulate various micronutrients, including iron, zinc and provitamin A (McGuire, 2015; Cakmak and Kutman, 2017; Wakeel et al., 2018; Zheng et al., 2020; Rehman et al., 2021). Vitamin A deficiency (VAD) is the major reason for childhood blindness and mortality, particularly impacting preschool children (West and Darnton-Hill, 2008; Greiner, 2013). To combat VAD and compensate for the scarcity of vitamin A in animal-derived products, several provitamin A biofortified crops, golden crops, have been generated by using metabolic engineering, including Golden Rice as the best-known example (Ye et al., 2000; Giuliano, 2017; Zheng et al., 2020).

**Figure 2:**
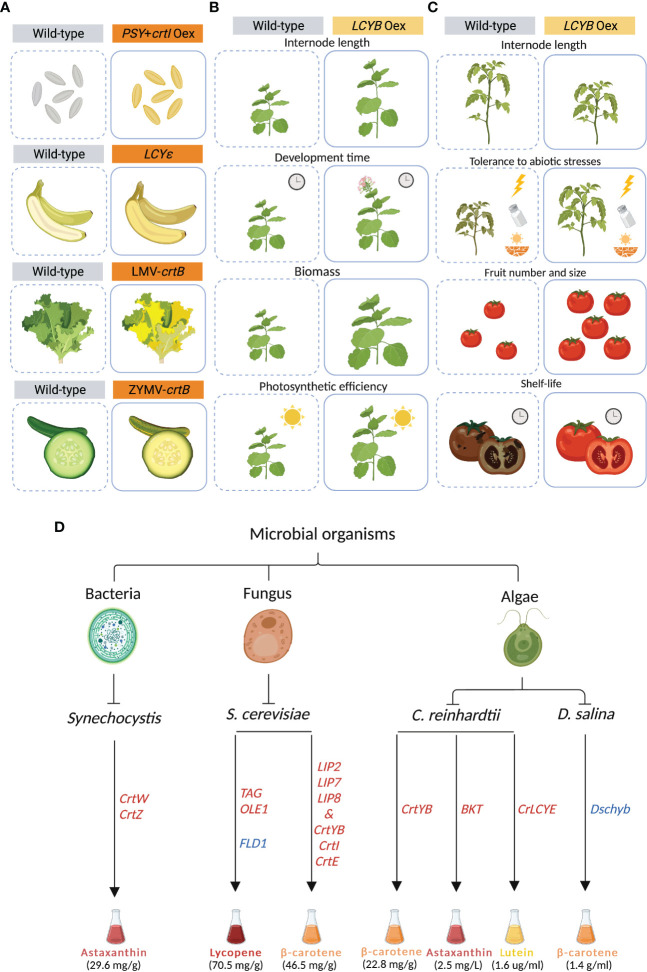
Synthetic carotenoid biosynthesis in plants and microorganisms. **(A)** Golden Rice was generated by co-expressing the *phytoene synthase* (*PSY*) and the bacterial *crtI* in the carotenoid-free rice endosperm. In Cavendish banana, *lycopene epsilon-cyclase* (*LCYE*), was mutated, leading to an increase in β-carotene. In lettuce (*Lactuca sativa*) and zucchini (*Cucurbita pepo*), the virus-mediated overexpression of the bacterial *crtB* resulted in carotenoid accumulation in leaves and fruits, respectively. **(B)** The overexpression of *lycopene-β-cyclase* (*LCYB*) gene in tobacco (*Nicotiana tabacum* cv. Xanthi) resulted in increased internode length, accelerated development and flowering time, and enhanced plant biomass and photosynthetic efficiency. **(C)** The overexpression of *lycopene-β-cyclase* (*LCYB*) gene in different cultivars of tomato (*Solanum lycopersicum*) altered internode length, and increased tolerance to abiotic stresses, number and size of fruits, and fruit shelf-life. **(D)** Different strategies for carotenoid mass production in *Synechocystis*, *Yarrowia lipolytica*, *Saccharomyces cerevisiae*, *Chlamydomonas reinhardtii*, and *Dunaliella salina*. Overexpressed and suppressed genes are depicted in red and blue, respectively. Figure was designed in Biorender.

One of the main strategies that has been pursued to increase carotenoid content is the “push” approach, which relies on enhancing the carotenoid metabolic flux by over-expressing one or more biosynthetic enzymes (Zheng et al., 2020). Since PSY catalyzes a rate-limiting step, it has been a major target for genetic engineering in many plants, including rice, tomato and cassava (Paine et al., 2005; Fraser et al., 2007; Paul et al., 2017; Yao et al., 2018; Strobbe et al., 2018). *PSY* was constitutively over-expressed for the first time in tomato (*Solanum lycopersicum*), resulting in dwarf plants, likely due to a depletion of the precursor GGPP that also feeds gibberellin biosynthesis (Fray et al., 1995). To by-pass unwanted side effects, constitutive-expression strategies were replaced by using tissue-specific promoters. In canola (*Brassica napus*), the bacterial *PHYTOENE SYNTHASE* (*crtB*) was over-expressed under the control of a seed-specific promoter, generating orange embryos that reached up to a 50-fold increase in carotenoid content (Shewmaker et al., 1999). Co-expression of *PSY* from daffodil (*Narcissus pseudonarcissus*) under an endosperm specific promoter and *crtI* from *Erwinia uredovora* (formerly *Pantoea ananatis*) under 35-S promoter in rice (*Oryza sativa*) led to Golden Rice, that accumulated β- and α-carotene, zeaxanthin and lutein, which are responsible for the yellow color of the grain (Ye et al., 2000; Datta et al., 2003; Strobbe et al., 2018). Later, “Golden Rice 2 (GR2)” was generated by replacing the daffodil-derived *PSY* with the more efficient ortholog from maize, increasing carotenoid content up to 23-fold compared to the former “Golden Rice” (**Figure 2A**) (Paine et al., 2005). Additionally, *PSY* was introduced in other crops, including potato, kiwi, and cassava, hence generating multiple golden crops (Al-Babili and Beyer, 2005; Diretto et al., 2007; Ampomah-Dwamena et al., 2009; Welsch et al., 2010).

More recently, new strategies have been developed to increase carotenoid content in several crops (**Table 1**). Carotenoid biofortification has been successfully achieved using CRISPR-mediated genome editing by specific gene/locus targeting (Zheng et al., 2021). A CRISPR-Cas9-based method was developed to generate a biofortified, marker-free rice line (Dong et al., 2020). First, a mutant screen analysis facilitated the identification of a specific “safe harbor” in the rice genome, in which the introduction of DNA is expected to not cause any side effect. The “safe harbor” locus was then used for introducing the GR2 carotenoid-biosynthesis cassette. The CRISPR cassette was then segregated by back-crossing, leading to a marker-free line that shows the golden phenotype. In another example, CRISPR-Cas9 was used to knock-out *LCYE* in Cavendish banana by targeting its fifth exon (Kaur et al., 2020). Here, the presence of several indels within the *LCYE* gene led to an up to 6-fold increase in β-carotene content (**Figure 2A**).

**Table 1:** Recent advances in carotenoid biofortification and metabolic engineering.

Plants	Strategy	Phenotypic change	Reference(s)
Rice	GR2 cassette inserted in “safe harbor” (CRISPR)	Orange embryos	Dong et al., 2020
Tobacco	*crtB*, c*rtE* and c*rtI* transiently expressed together with a truncated version of HMGR	Yellow leaves	Andersen et al., 2021
	Arabidopsis *VDE*, *ZEP* and the *PsbS* overexpression	Accelerated response to fluctuating light	Kromdijk et al., 2016
	Carrot *LCYB* overexpression	Longer internodes, early flowering, accelerated development, increased biomass, yield, photosynthetic efficiency and enhanced abiotic stress tolerance	Moreno et al., 2021b
	(RNAi) *NtLCYB*	Impaired growth, photosynthesis and reduced pigment content	Kössler et al., 2021
Tomato	*LCYB* overexpression	Increased growth, yield, fruit dry weight and shelf-life, and abiotic stress tolerance	Mi et al., 2022
Banana	*LCYE* knock-out (CRISPR)	Orange fruits	Kaur et al., 2020
Soybean	Arabidopsis *VDE*, *ZEP* and *PsbS* overexpression	Up to 33% increase in yield	De Souza et al., 2022
Lettuce, zucchini, tobacco leaves	*crtB* overexpression	Yellow leaves	Llorente et al., 2020
Arabidopsis	*ARC3* overexpression in *OR^His^* background	Orange callus	Sun et al., 2020

Transient expression allows the expression of genes without the integration in the genome, generating results in a faster way compared to stable transgenic plants (Page et al., 2019). Transient expression systems are a promising tool for enriching carotenoids, including provitamin A, in green tissues at a specific developmental stage without interfering with normal plant growth and development (Rodríguez-Concepción and Daròs, 2022). In lettuce (*Lactuca sativa*) and zucchini (*Cucurbita pepo*), the virus-mediated expression of *crtB* induced the re-organization of internal plastid structures, which resulted in the differentiation of chloroplasts into chromoplasts and conferred a yellow color to the fruits (**Figure 2A**) (Llorente et al., 2020). In tobacco (*Nicotiana tabacum*), the high phytoene levels triggered by the transient expression of *crtB* interferred with chloroplast functions by lowering their photosynthetic efficiency and activating an endogenous developmental program enabling complete chloroplast-chromoplast switch and increasing carotenoid storage capacity. In addition, the overexpression of this gene stimulated the accumulation of β-carotene and lutein in the agroinfiltrated leaves, conferring a yellow leaf phenotype.

ORANGE (OR) proteins have been shown to post-transcriptionally regulate PSY activity during carotenoid biosynthesis and promote chromoplast biogenesis (Lopez et al., 2008; Chayut et al., 2017; Osorio, 2019). The expression of *OR* gene enhanced carotenoid accumulation in several crops by triggering the formation of chromoplasts containing carotenoid sequestering structures (Lopez et al., 2008; Park et al., 2015; Yazdani et al., 2019). However, only one to two big chromoplasts were found in the cells of the orange cauliflower *or* mutant (Paolillo et al., 2004). A recent study demonstrated that the OR^His^ variant, which contains a single histidine substitution (“golden SNP”), interacts with two proteins involved in plastid division (PARC6 and ARC3), thus limiting the chromoplast number (Sun et al., 2020). The overexpression of ARC3 in a mutated *OR^His^* Arabidopsis line increased carotenoid accumulation up to 85% compared to the control.

One of the main challenges of reaching a high-carotenoid accumulation in plants is the tight pathway regulation in plastids, which is achieved by metabolic feedback and feedforward signaling (Cazzonelli and Pogson, 2010). Hence, enabling carotenoid production based on cytosolic mevalonate-derived isoprenoid precursors is a very interesting option that has been recently explored (Andersen et al., 2021). A viral vector that co-express *CrtE* (*GGPP synthase*), *crtB*, and *crtI* genes was inoculated in tobacco leaves, thus successfully stimulating lycopene accumulation outside the chloroplast and turning the leaves yellow (Majer et al., 2017). This strategy was then optimized in *Agrobacterium tumefaciens* by the additional use of a truncated version of the enzyme hydroxymethylglutaryl CoA reductase (HMGR), which boosted the mevalonate (MVA) pathway in the cytosol and provided more GGPP precursors (Andersen et al., 2021). While cytosolic phytoene remained more bioaccessible, lycopene was stored in less accessible cytosolic crystalloids. Interestingly, the content of extra-plastidial carotenoids was similar to that of endogenous chloroplast carotenoids, which explains the orange coloration of infected leaves (Andersen et al., 2021).

The employment of new technologies, such as CRISPR-based gene-editing tools and viral vectors, is expected to open new prospects in carotenoid biofortification in the near future. Indeed, genome editing tools may enable precise manipulation of regulatory genes governing the carotenoid biosynthetic pathway. As shown by Dong et al. (2020), CRISPR technologies also allow to accommodate transgenes at specific loci. However, this approach is limited by the low efficiency of donor delivery and plant transformation, which makes the biofortification of some crops, e.g. pearl millet, a difficult task. A further novel approach is the generation of non-plastid sinks to redirect carotenoid biosynthesis and boost both the production and storage of carotenoids in green vegetables. Given the promising results obtained with installing transient carotenoid biosynthesis in the cytosol, it can be expected that generating corresponding, stably transformed plants may make significant contributions to biofortification (Morelli and Rodriguez-Concepcion, 2022).

## Carotenoid metabolic engineering beyond biofortification

Climate change and extreme weather events directly impact agriculture and crop production (Raza et al., 2019; Pareek et al., 2020). According to food demand predictions, the current increase in crop yields is insufficient to compensate for the losses due to global warming (Kromdijk et al., 2016). The emerging combination of several abiotic stresses, such as increasing drought, extreme temperatures, and high UV irradiation, directed researchers to better understand stress-resistance processes towards developing stress-tolerant crops (Pereira, 2016). Carotenoid biosynthesis and accumulation seem to positively impact the resistance of plants to different types of environmental stress, such as high-light, increased temperature and drought (Uarrota et al., 2018; Kim et al., 2018; Swapnil et al., 2021). Accordingly, mutants of photosynthetic organisms with reduced carotenoid content are more susceptible to photo-oxidation (Ramel et al., 2013). Therefore, modifying carotenoid biosynthesis represents a promising option for developing resilient crops.

Xanthophyll cycle plays a major role in protecting the photosynthetic apparatus from photo-oxidative stress (Latowski et al., 2011). In the Arabidopsis *lut2 npq2* double mutant, the xanthophylls neoxanthin, violaxanthin, antheraxanthin, and lutein were all replaced by zeaxanthin (Havaux et al., 2004). This conversion resulted in an enhanced tolerance against photo-oxidation and in a phenotype similar to that of high-light-acclimated leaves. Moreover, a different study showed that doubling the size of the xanthophyll pool led to increased resistance to high light and high-temperature conditions (Johnson et al., 2007). This result is probably due to a reduction of the ROS-induced lipid peroxidation in presence of enhanced zeaxanthin content.

In recent years, metabolic engineering of carotenoids succeeded in enhancing crop yield and fitness (**Table 1**). For instance, the expression of the Arabidopsis *VDE*, *ZEP*, and the *PSII subunit S* (*PsbS*) in tobacco leaves enabled accelerated response to fluctuating light, thus enhancing the efficiency of CO_2_ assimilation in the shade by 14% and increasing plant dry weight biomass up to 15% (Kromdijk et al., 2016). Interestingly, opposite results were observed when this strategy was applied in Arabidopsis (Garcia-Molina and Leister, 2020). The same construct was recently introduced in soybean (*Glycine max*), leading to a ~33% increase in yield (De Souza et al., 2022). Thus, species-specificity of the impact of foreign gene expression in crops and model plants requires further analysis.

Recently, genetic manipulation of the *LCYB* gene has been shown as a promising strategy for crop improvement beyond biofortification (Moreno and Al-Babili, 2022). A single-gene strategy has been applied in carrot (*Daucus carota*) and in tobacco (*Nicotiana tabacum* cv. *Xanthi*) where the expression of the carrot *LCYB1* gene led to changes in plant growth, architecture, and development (Moreno et al., 2013; Moreno et al., 2016; Moreno et al., 2020). In tobacco, the alteration in carotenoid and phytohormone composition triggered several phenotypes, including longer internodes, early flowering, accelerated development, increased biomass, yield, and photosynthetic efficiency. Interestingly, the transgenic tobacco lines also showed enhanced abiotic stress tolerance (**Figure 2B**) (Moreno et al., 2021b). Moreover, the RNA interference (RNAi) *NtLCYB* tobacco lines showed impaired growth and photosynthesis, and reduced pigment content and plant variegation (Kössler et al., 2021). A similar approach was applied to different tomato cultivars where the overexpression of *LCYB*, from plant or bacterial origin, modulated carotenoid, apocarotenoid and phytohormones patterns, resulting in several phenotypes, including biomass partitioning and altered growth, and an improvement in fruit yield and shelf-life, and abiotic stress tolerance (**Figure 2C**) (Mi et al., 2022).

These promising results proved that the alteration of the carotenoid pathway, *via LCYB* genetic manipulation, can directly impact several interconnected metabolic networks, including the biosynthesis of phytohormones and signaling molecules affecting several plant traits, such as growth, yield, and stress tolerance, which are key for crop improvement. It would be interesting to investigate how manipulation of well-characterized carotenoid metabolic genes can influence the phenotype and other desirable traits in cereals, such as rice and pearl millet, beyond biofortification. Future research should also focus on the impact of modifying carotenoid content on rhizospheric interactions and root symbiotic associations, i.e. AM fungi, and explore the possibility of improving beneficial interactions to generate crops with better performance.

## Metabolic engineering of carotenoid biosynthesis in microorganisms

In recent years, the demand for natural carotenoids has continuously increased with the rapidly growing food, pharmaceutical and cosmetic industry; thus, creating a need for natural sources for their mass production (Clugston, 2020; Zerres and Stahl, 2020; López et al., 2021). The production of carotenoids from bacteria has received wide attention due to their short life cycle and high productivity (Sajjad et al., 2020). Carotenoids extracted from bacteria are safe for humans as those obtained from traditional sources such as plants or chemical synthesis (Numan et al., 2018). Indeed, there is a wide range of applications for bacterial carotenoids, including the using of *Brevibacterium linens* in the fermentation of Limburger and Port-du-Salut cheeses, which is responsible for the characteristic color of these dairy products (Guyomarc’h et al., 2000). In addition, astaxanthin produced in *Mycobacterium lacticola* is used for fish feeding, due to its antioxidant activity and to obtain the red color that attracts consumers (Kirti et al., 2014).

A recent study showed that engineered cyanobacteria can produce valuable carotenoids such as astaxanthin and lutein, which exert beneficial biological activities, such as being antioxidants and important colorants (Honda, 2022). In particular, the model cyanobacterium *Synechocystis* sp. PCC 6803 is able to divert 50% of its carbon flux to the synthesis of carbon-containing compounds (Angermayr et al., 2016). To produce high levels of astaxanthin from CO_2_ in this cyanobacterium, the key enzymes *crtW* and *crtZ* were co-expressed, and the carbon flux was redirected towards the endogenous MEP pathway by increasing precursor availability, leading to the accumulation of up to 29.6 mg/g (dry weight) of astaxanthin (**Figure 2D**) (Diao et al., 2020).

Utilizing fungal organisms is considered as one of the most advantageous ways for mass production of carotenoids (Wang et al., 2021b). Beaker yeast, *Saccharomyces cerevisiae*, has a large cell size, can tolerate distinct growth conditions, e.g. low temperature, and possesses segmented organelles, thus making it a promising host to install carotenoid production (Madhavan et al., 2022). To improve the production of lycopene in *S. cerevisae*, key genes related to fatty acid synthesis and triacylglycerol (TAG) production were overexpressed together with a fatty acid desaturase (OLE1) that forms unsaturated fatty acids. In addition, a gene (*FLD1*) encoding Seipin that regulates lipid-droplet size was deleted. The resulting *S. cerevisiae* strain showed a 25% increase in lycopene accumulation, compared to the original high-yield strain (**Figure 2D**) (Ma et al., 2019). Overexpressing three lipase-coding genes (*LIP2*, *LIP7* and *LIP8*) from *Yarrowia lipolytica* together with *PHYTOENE SYNTHASE/LYCOPENE CYCLASE* (*crtYB*), *crtI* and *GERANYLGERANYL DIPHOSPHATE SYNTHASE* (*crtE*) cloned from the red yeast *Xanthophyllomyces dendrorhous* is a further promising strategy that resulted in 46.5 mg/g (dry weight) of β-carotene accumulation, i.e. 12-fold higher than the analogous strain lacking lipase expression (**Figure 2D**) (Fathi et al., 2021).

The model yeast, *Y. lipolytica*, is one of the widely used species in food industry. *Y. lipolytica* possesses a high concentration of acetyl-CoA, which is essential to enhance the production of β-carotene (Gao et al., 2017; Zhang et al., 2018). A novel study described two different effective approaches in *Y. lipolytica* to overcome the LCYB substrate inhibition, which represents an undesired regulatory mechanism triggered by high substrate concentration. (Ma et al., 2022). Firstly, by using a structure-guided protein design, the single variant mutation Y27R was generated, which completely removed the substrate inhibition without reducing the enzymatic activity. Then, a GGPPS-mediated restrictor was constructed, which regulates the lycopene formation rate, thus limiting the carbon flux through the carotenoid biosynthesis pathway and, consequently, alleviating substrate inhibition. The final engineered strain led to 39.5 g/L of β-carotene production in *Y. lipolytica*.

Microalgae are a diverse group of photosynthetic organisms found in aquatic habitats, which provide new options for enhanced production of carotenoids, due to their low cultivation costs, simplicity and rapid growth rate. They are a common host used by the pharmaceutical industry for the production of naturallly coloring pigments, besides being a source for biofuels (Varela et al., 2015; Khan et al., 2018; Novoveská et al., 2019; Sahoo et al., 2020). *Dunaleilla salina, Haematococcus pluvialis, and Chlorella vulgaris* are examples for microalgae rich in β-carotene, astaxanthin, lycopene, lutein, and zeaxanthin content (Xu et al., 2018; Velmurugan and Muthukaliannan, 2022). *Chlamydomonas reinhardtii* is one of the fastest-growing microalgae, which has been used for the production of carotenoids, including β-carotene and lutein (Rathod et al., 2020). In a recent study, it was shown that the expression of the bifunctional *PHYTOENE-β-CAROTENE SYNTHASE* (*crtYB*) from *X. dendrorhous* in *C. reinhardtii* resulted in a 72% and 83% increase in β-carotene and lutein content, respectively, when exposed to “short duration on high-light” (SD-HL) (**Figure 2D**) (Rathod et al., 2020). In a different study, overexpression of a codon-optimized native *β-CAROTENE KETOLASE* (*BKT*) in *C. reinhardtii* pushed the conversion of more than 50% of total carotenoids into astaxanthin (**Figure 2D**) (Perozeni et al., 2020). In addition, it was shown that the overexpressing native *LCYE* in *C. reinhardtii* enhanced the lutein accumulation by up to 2.6-fold through increasing the conversion of lycopene into α-branch carotenoids, which eventually increased lutein production (1.6 ug/ml of culture) (**Figure 2D**) (Tokunaga et al., 2021).

Additionally, a recent study demonstrated the impact of overexpressing a mutated and wild type *ORANGE* (*OR*) in *C. reinhardtii* under the control of a strong light-inducible promoter (Yazdani et al., 2021). The mutated *CrOR^His^* strain contained up to 1.6-fold and 3.2-fold enhanced total carotenoid content, compared to the wild-type *CrOR* overexpressing and the mock line, respectively. In *Dunaliella salina*, a CRISPR-Cas technique was used to target the 1^st^ and 3^rd^ exons of the *β-CAROTENE HYDROXYLASE* (*Dschyb*) gene, which significantly enhanced the β-carotene level, reaching about 1.4 g/ml (Hu et al., 2021) (**Figure 2D**).

Taken together, microorganisms are promising sources for enhanced production of carotenoids for research and industrial use. Identifying new genes and enzymes from different microbial sources will enlarge our toolkit for carotenoid mass-production, needed to expand the variety of products and to meet the increasing demand of growing industry in the area of food, cosmetics and pharmacy.

## Concluding remarks

The carotenoid metabolic pathway has been studied extensively and manipulated over the years to generate crops with improved carotenoid content and productivity. The advent of new gene-editing techniques, such as CRISPR, allows precise and targeted editing of carotenoid-related genes, thus avoiding the side effects conferred by the random insertion of the transgenic cassette. However, despite CRISPR-based tools proved to be highly efficient in generating precise deletions and single-nucleotide substitutions, gene knock-ins are still very difficult to achieve in many crops. In fact, donor insertions still have a very low efficiency and several limitations, such as the high number of off-targets and the limited donor length. Further efforts are needed to develop new CRISPR-based strategies to efficiently obtain cisgenic plants with native gene knock-in. For instance, gene duplication approaches can be used to obtain gene overexpression without the introduction of a foreign donor. An alternative approach could be the deregulation of carotenoid key genes by swapping or disturbing their native promoters (Lu et al., 2021). However, the employment of “classical” transgenic approaches is expected to remain indispensable if carotenoid biofortification requires the introduction of phytoene synthesis and its multi-step desaturation to drive carotenoid biosynthesis in carotene-free tissues, such as rice endosperm, or cellular compartments, i.e. cytoplasm.

With respect to our knowledge on carotenoid and apocarotenoid metabolism, the deployment of stress, light, and chemically inducible promoters regulating the expression of carotenoid biosynthesis and catabolic genes, and suitable analytical tools could provide new insights in carotenoid metabolism. Developing such new strategies supported by innovative and versatile analytical techniques and methodologies is crucial for better elucidating carotenoid and apocarotenoid metabolism, signaling and regulation and, hence, for developing the crops of the future with a higher yield and adaptation.

## Author contributions

AS: wrote the abstract, sections Carotenoid biosynthetic pathway in plants and microorganisms, Carotenoid biofortification in plants, Carotenoid metabolic engineering beyond biofortification, part of Metabolic engineering of carotenoid biosynthesis in microorganisms, and prepared **Figures 1**, **2** (with LA and YA). LA: wrote part of section 4. JM, YA and SA-B: extensively edited the provided manuscript and provided supervision. All authors contributed to the article and approved the submitted version.
